# PD-1 induces autophagy via the PI3K/AKT/FoxO1 pathway to promote infectious bursal disease virus replication

**DOI:** 10.3389/fimmu.2025.1585012

**Published:** 2025-07-07

**Authors:** Qiuyu Zhang, Feng Yue, Guopeng Sun, Liwei Jiang, Peng Li, Yanping Zhu, Zhike Liu, Yangzhao Zhu, Ruiyan Niu, Hua He, Zilong Sun, Xuannian Wang

**Affiliations:** ^1^ College of Veterinary Medicine, Henan Agricultural University, Zhengzhou, China; ^2^ College of Veterinary Medicine, Shanxi Agricultural University, Jinzhong, China; ^3^ Longhu Laboratory, Zhengzhou, China; ^4^ College of Biological Engineering, Xinxiang University, Xinxiang, China; ^5^ College of Animal Science and Veterinary Medicine, Xinyang Agriculture and Forestry University, Xinyang, China; ^6^ College of Animal Science and Veterinary Medicine, Henan Institute of Science and Technology, Xinxiang, China

**Keywords:** infectious bursal disease virus, PD-1, autophagy, PI3K/AKT, FoxO1, viral replication

## Abstract

**Introduction:**

Autophagy is an important process in host cell responses to viral replication and spread, including those against infectious bursal disease virus (IBDV). Programmed death-1 (PD-1) is a known immunoinhibitory receptor, and its expression causes immune dysfunction in B lymphocytes, resulting in increased progression of immunosuppressive diseases. However, the role of PD-1 in autophagy during IBDV infection remains unclear.

**Methods:**

We investigated the mechanism by which chicken PD-1 regulates autophagy during IBDV infection.

**Results:**

IBDV infection enhanced PD-1 expression in chicken tissues and DT-40 cells. Subsequent interaction analyses revealed that PD-1 interacted only with the viral protein VP2 to enhance the IBDV replication in DT-40 cells. PD-1 overexpression significantly increased IBDV-induced autophagy, whereas silencing of PD-1 had the opposite effect in IBDV-infected DT-40 cells. Furthermore, PD-1 enhanced the activation of FoxO1 via the PI3K/AKT pathway. Finally, we demonstrated that autophagy is critical for role of PD-1 in regulating VP2 protein expression and IBDV titers.

**Discussion:**

These findings present a novel mechanism wherein PD-1 induces autophagy by activating the PI3K/AKT/FoxO1 pathway to facilitate IBDV replication, providing a new avenue in developing universal vaccine adjuvants for IBDV infection control.

## 1 Introduction

Infectious bursal disease (IBD) is a highly contagious and immunosuppressive infectious disease in young chickens caused by IBD virus (IBDV) belonging to the genus *Avibirnavirus* of the family *Birnaviridae* (1). IBDV causes a massive destruction of B lymphocytes in the bursal of Fabricius (BF), resulting in increased susceptibility to other pathogenic infections and reduced vaccination efficacy (2). IBDV is a non-enveloped virus with a double-stranded RNA genome, which includes segments A and B, VP1, VP2, VP3, VP4, and VP5 (3). Among these, VP1, VP2, and VP3 are structural proteins essential for virus replication and pathogenicity (4–6). IBDV has evolved complex mechanisms to manipulate host immune responses through virus-host interactions, thereby promoting its survival and replication (7). However, the pathogenic mechanisms of IBDV infection remain largely unclear.

PD-1, as a host immunoinhibitory factor, is primarily expressed on the membranes of activated T and B cells. Lymphocytes are crucial for host immune response, and play a key role in defending against and controlling various viral infections (8, 9). The interaction between PD-1 and PD-L1 leads to apoptosis and functional exhaustion of lymphocytes during chronic viral infections, such as those caused by Marek’s disease virus, hepatitis B virus, and bovine leukemia virus (10–12). Activation of PD-1 is reported to negatively regulate B-cell activation and proliferation (13). Furthermore, our previous findings also showed that IBDV infection enhances the protein expressions of PD-1 and PD-L1 and the functional exhaustion of B lymphocytes to support its replication in chicken B lymphocytes (14). Importantly, autoimmune diseases are associated with the dysregulation of autophagy machinery, contributing to enhanced cell death induced by apoptosis and autophagy (15).

Autophagy is a crucial process that regulates intracellular homeostasis by degrading cellular components (16). Various studies have shown that some viruses have evolved different mechanisms to interfere with autophagy and facilitate their own propagation (17). For example, influenza A virus, Newcastle disease virus, and avian reovirus induce autophagosomes formation and utilize autophagy machinery to promote viral protein synthesis (18–20). IBDV infection promotes autophagosome-lysosome formation and leads to immune function disorders, which in turn facilitate virus maturation and release (21). IBDV is believed to utilize the autophagy machinery to regulate cell functions and enhance its replication. However, the detailed functional role of PD-1 in IBDV-induced autophagy remains unclear. Therefore, exploring the relationship between autophagy and host proteins will help understand the infection mechanisms of IBDV and further develop prevention and control strategies.

The PI3K/AKT pathway is essential for regulating various cellular processes, including proliferation, apoptosis, autophagy, and protein synthesis (22). Furthermore, activation of the PI3K/AKT pathway has been shown to inhibit autophagy in mammalian cells (23, 24). FoxO1 is considered a crucial mediator that plays a significant role in regulating autophagy by binding to the promoters of autophagy-related proteins in specific cell types such as macrophages and lymphocytes (25, 26). Further, FoxO1 activity is regulated by the PI3K/AKT pathway in germinal center B cells (27). Our previous study unveiled that the PI3K/AKT pathway was inactivated after IBDV infection (14). However, whether the activation of PI3K/AKT/FoxO1 pathway involvement regulates autophagy in IBDV-infected B lymphocytes remains unclear.

Here, we firstly investigated the mechanism by which chicken PD-1 regulates autophagy during IBDV infection.

## 2 Materials and methods

### 2.1 Cells and viruses

The chicken lymphoid cell line DT-40 was cultured in Dulbecco’s modified Eagle’s medium (DMEM) supplemented with 10% fetal bovine serum (FBS) and 5% chicken serum at 37 ˚C with 5% CO_2_ in a humidified incubator. The vvIBDV strain LX has been previously described (28).

### 2.2 Antibodies and reagents

Anti-HA mouse monoclonal antibodies (1:3000/1:800; cat# D191044) and anti-Myc rabbit polyclonal antibodies (1:1500/1:500; cat# D110006) were purchased from Sangon Biotech (Shanghai, China). Anti-β-actin (1:3000; cat# T40104S), anti-Myc (1:3000; cat# M20002), anti-LC3B (1:1000; cat# T55026), anti-VP2 (1:1000; cat# M032148S), anti-ATG5 (1:1000; cat# T55766), anti-p-PI3K (1:1000; cat# TA4371), anti-PI3K (1:1000; cat# TA5121), anti-p62 (1:1000; cat# P76367R1S), anti-p-AKT (1:1000; cat# TA0016), anti-AKT (1:1000; cat# PA1036), and horseradish peroxidase-conjugated anti-mouse/rabbit IgG antibodies were purchased from Abmart (Shanghai, China). Anti-FoxO1 (1:1000; cat# bs-9439R and anti-p-FoxO1 (1:1000; cat# bs-3142R) antibodies were purchased from Bioss (Beijing, China). Anti-chicken PD-1 (1:100) mouse monoclonal antibodies were generated in our laboratory (14). NP-40 lysis buffer (Cat# P0013F) and anti-HA magnetic beads (Cat# P2121) were purchased from Beyotime (Shanghai, China). LY294002 (Cat# HY-10108), 3-methyladenine (3-MA, cat# HY-19312), and SC79 (Cat# HY-18749) were dissolved in dimethyl sulfoxide (DMSO) and purchased from MedChemExpress (Princeton, NJ, USA).

### 2.3 Virus infection

In all, 30 SPF chickens (3 weeks old) were purchased from Yuanfa (Xinxiang, China) and randomly divided into two groups (infected and non-infected). Each chicken was infected with either 200 μL of 10^5^ ELD_50_ of vvIBDV or PBS. Three days post-infection (dpi), all chickens were euthanized and the chicken tissues were harvested for further analysis. DT40 cells were seeded at 10^7^ cell in 6-well cell culture plates for 24 h and then inoculated them with IBDV at a multiplicity of infection (MOI) of 1 for the indicated times. Cells or culture supernatants were collected for further analysis, as required.

### 2.4 Quantitative real-time PCR analysis

Total RNA was extracted from different chicken tissues and reverse transcribed into cDNA. qRT-PCR was performed using a QuantStudio 5 Real-Time PCR System. The relative expression of *PD-1* was normalized to that of *β-actin*. The primers used were *PD-1* (F: 5′-GGACTACGGTGTGCTGGAGTT-3′; R: 5′-TCTTTCCTCGCTCTGGTGTG-3′) and *β-actin* (F: 5′- TATTGCTGCGCTCGTTGTTGAC-3′; R: 5′-GATACCTCTTTTGCTCTGGGCTTC-3′).

### 2.5 Plasmid and small-interfering RNA

Plasmids pXJ40-myc-VP1, pXJ40-myc-VP2, pXJ40-myc-VP3, pXJ40-myc-VP5, pXJ40-myc-PD-1, pXJ40-HA-PD-1, pEGFP-HA-PD-1, and pEGFP-HA-VP2 were constructed using the specified restriction enzyme sites and PCR methods. Empty vectors pXJ40-myc, pXJ40-HA and pEGFP-N1 were stored in our laboratory. Plasmids mRFP-GFP-LC3, pXJ40-myc-VP4, and pEGFP-myc-FoxO1 were purchased from Synbiob (Tianjing, China). Chicken FoxO1 and PD-1 siRNAs and non-targeting siRNAs (siNC) were synthesized by GenePharma (Suzhou, China). The following primers were used for siRNA: siPD-1#1 (sense: GACUCAGGCUUCUACUACUTT antisense: AGUAGUAGAAGCCUGAGUCTT), siPD-1#2 (sense: GCAGAAACCAUCAAGUGGATT antisense: UCCACUUGAUGGUUUCUGCTT), siPD-1#3 (sense: GAAAGAGGCACAAGGAUGATT antisense: UCAUCCUUGUGCCUCUUUCTT), siFoxO1#1 (sense: GCGCCGACUUCAUCAGCAA antisense: UUGCUGAUGAAGUCGGCGC), siFoxO1#2 (sense: GGGCGACAGCAACAGUUCA antisense: UGAACUGUUGCUGUCGCCC), siFoxO1#3 (sense: CGACCUAGAACUAGCUCUA antisense: UAGAGCUAGUUCUAGGUCG), and siNC (sense: UUCUCCGAACGUGUCACGUTT antisense: ACGUGACACGUUCGGAGAATT).

### 2.6 Transfection

DT-40 cells in 6-well plates were grown to approximately 60–70% confluency and transfected them with 2 µg of the indicated plasmids using Lipofectamine 2000 (Invitrogen, Carlsbad, CA, USA), as per the manufacturer’s instructions. The siRNAs were transfected into DT-40 cells using Lipofectamine RNAiMAX transfection reagent (Invitrogen, Carlsbad, CA, USA), according to the manufacturer’s instructions. At 24 h post-transfection, the transfected cells were infected with IBDV at MOI 1 for 24 h or treated with small-molecule compounds for the indicated times.

### 2.7 Western blotting

Western blotting was conducted as previously described method (29). The cell lysates were separated by 8–12% SDS-PAGE, followed by transfer to nitrocellulose membrane. After blocking with 5% skim milk powder the membranes were incubated overnight at 4°C with the indicated monoclonal or polyclonal antibodies. After washing thrice with PBST, the membranes were incubated with the corresponding secondary antibodies at room temperature. Finally, the signals from the membrane blots were scanned on the GE Amersham Imager 600 system (GE, Chicago, IL, USA). The intensities of the target proteins were quantified using ImageJ software version 5.0.

### 2.8 Co-immunoprecipitation

Co-immunoprecipitation (Co-IP) assays were conducted as previously described method (1). Briefly, DT-40 cells were co-transfected with the indicated plasmids for 24 h. Total proteins were lysed using an NP-40 lysis buffer containing phenylmethanesulfonyl fluoride. The cell lysate supernatant was incubated with the indicated antibodies or anti-HA magnetic beads. The resulting supernatants were removed by centrifugation at 3,000 × *g* for 3 min and subsequently used for western blotting analysis.

### 2.9 Virus titration

The IBDV titer was determined by measuring 50% egg lethal dose (ELD_50_) in eggs. Supernatants from IBDV-infected DT-40 cells were serially diluted with PBS. Then, 9-day-old SPF eggs obtained from Boehringer Ingelheim (Shanghai, China) were inoculated with 100 μL of diluted virus sample in the chorioallantoic membrane. After 7 days of inoculation ELD_50_ was calculated using the Reed-Muench method (30).

### 2.10 Indirect immunofluorescence staining

An indirect immunofluorescence assay was conducted as previously described method (6). Briefly, the BF tissue or DT-40 cells were fixed in 4% paraformaldehyde and permeabilized with 0.2% Triton X-100. Then, nonspecific binding was blocked using 3% bovine serum albumin. The processed sections or cells were incubated with the specified primary antibodies and corresponding secondary antibodies. Cell nuclei were stained with 4′6-diamidino-2-phenylindole (DAPI). Fluorescence images were acquired using an Eclipse C2 confocal laser-scanning microscope (Nikon, Tokyo, Japan).

### 2.11 Statistical analysis

Statistical analyses were conducted using the GraphPad Prism 8.0 software. All data are presented as the mean ± standard deviation (SD) from at least three independent experiments. Differences between groups were calculated using a Student’s t-test. Statistical significance was set at P < 0.05 (* P < 0.05 and ** P < 0.01).

## 3 Results

### 3.1 IBDV infection promotes PD-1 expression

**Figure 1:**
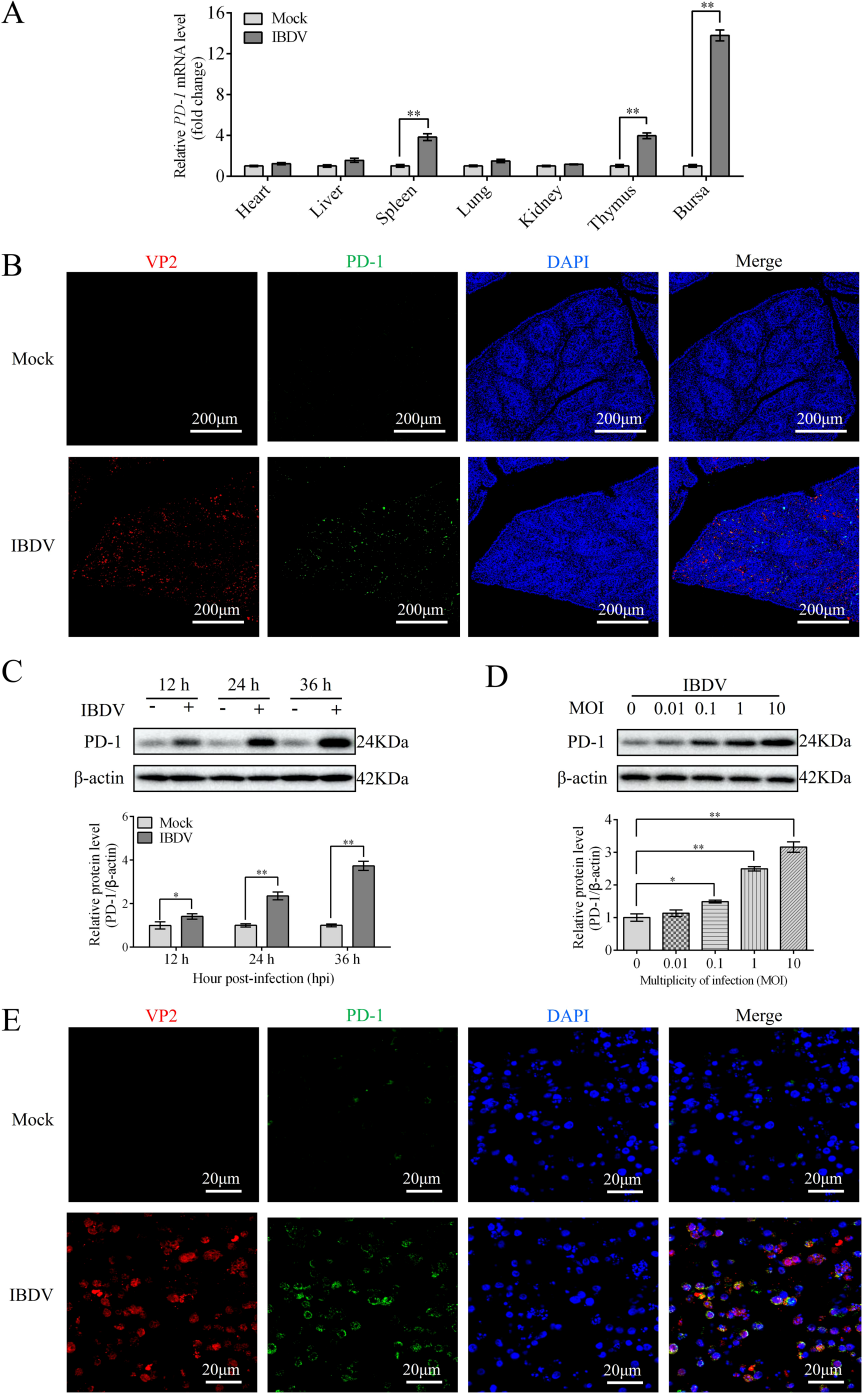
PD-1 is upregulated upon IBDV infection both *in vivo* and *in vitro*. Three-week-old SPF chickens were infected with 200 μL of 10^5^ ELD_50_ of IBDV for 3 d **(A)** The mRNA level of PD-1 was assessed by RT-qPCR in IBDV-infected tissues. **(B)** The protein expressions of VP2 and PD-1 in BF were observed by confocal microscopy (red, IBDV VP2; green, PD-1; blue, nucleus). Scale bar: 200 μm. **(C)** Protein expression of PD-1 was detected using western blotting in DT-40 cells infected with MOI 1 of IBDV for indicated time. **(D)** Protein expression of PD-1 was detected using western blotting in DT-40 cells infected with IBDV at different MOIs for 24 h **(E)** The distribution of VP2 and PD-1 proteins were observed by confocal microscopy (red, IBDV; green, PD-1; blue, nucleus). DT-40 cells were infected with IBDV at an MOI of 1 for 24 h Scale bar: 20 μm. Data represented mean ± SD from three replicates. *P < 0.05 and **P < 0.01.

Our previous study showed that PD-1 levels were elevated in the BF of IBDV-infected chickens (14). To analyze the expression of PD-1 in other tissues of infected chickens, chickens were infected with IBDV. At 3 dpi, the mRNA expression of PD-1 were measured in different tissues including the heart, spleen, liver, kidneys, lungs, thymus, and bursa. qRT-PCR analysis showed that PD-1 mRNA expression was significantly upregulated in the spleen, BF, and thymus tissues compared to that in other tissues in IBDV-infected chickens (**Figure 1A**). Because the BF is the primary target organ of IBDV, it was investigated in more detail. Immunofluorescence results also showed a significant increase in PD-1 levels in the BF tissues of infected chickens compared to those in the mock group (**Figure 1B**). Western blotting results revealed that PD-1 protein expression was upregulated in IBDV-infected DT-40 cells in time- and dose-dependent manners compared to that in the controls (**Figures 1C, D**). Additionally, confocal microscopy revealed that the VP2 and PD-1 proteins were greatly expressed during IBDV infection (**Figure 1E**). These findings indicate that IBDV infection promotes the protein expression of PD-1 both *in vivo* and *in vitro*.

### 3.2 VP2 enhances PD-1 expression

**Figure 2:**
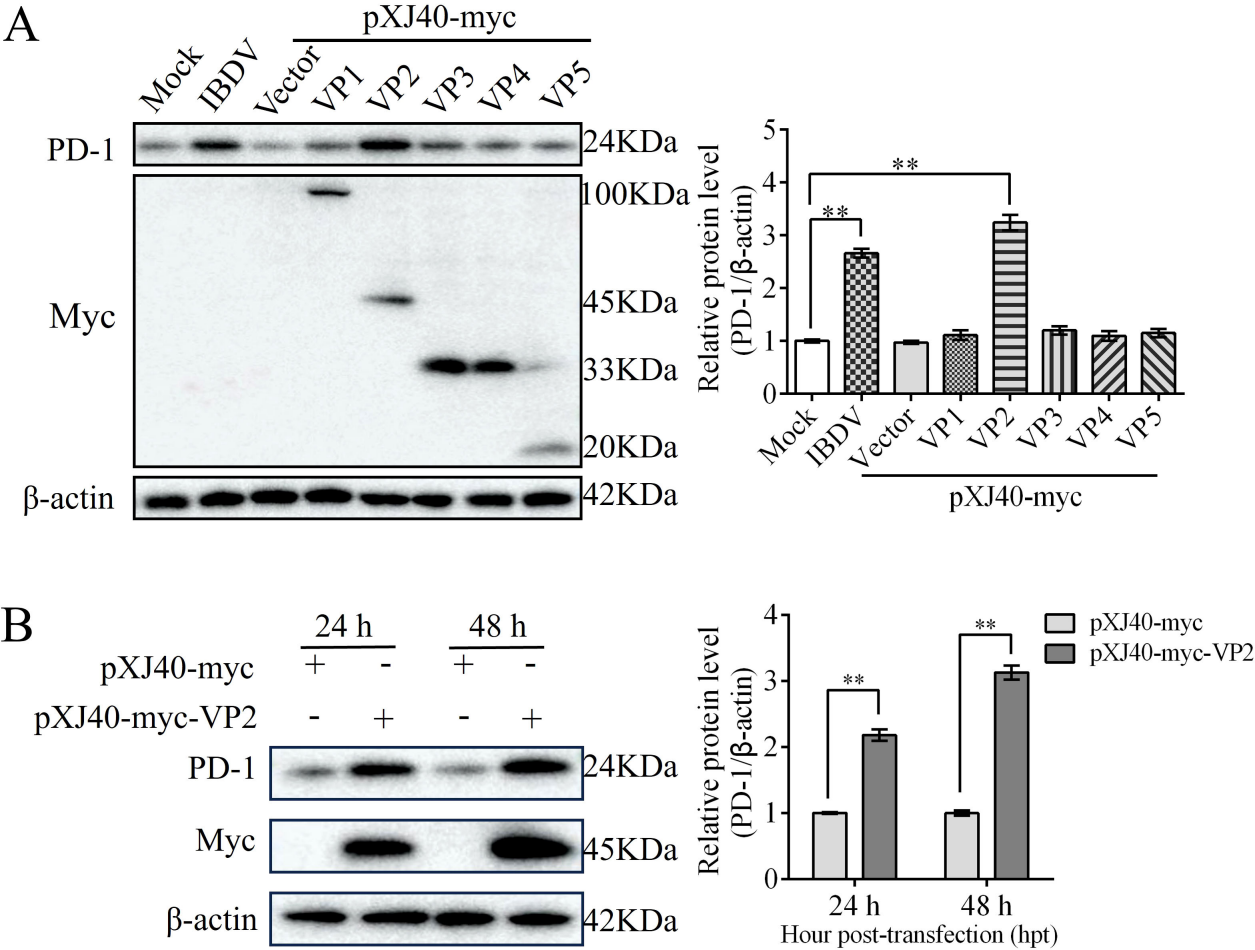
VP2 enhances PD-1 expression. **(A)** DT-40 cells were transfected with pXJ40-myc vector, pXJ40-myc-VP1, pXJ40-myc-VP2, pXJ40-myc-VP3, pXJ40-myc-VP4, and pXJ40-myc-VP5 for 24 h or infected with IBDV at an MOI of 1 for 24 h, followed using western blotting. **(B)** DT-40 cells were transfected with pXJ40-myc or pXJ40-myc-VP2 for 24 and 48 h, followed using western blotting. Data represented mean ± SD from three replicates. **P < 0.01.

To confirm which IBDV protein upregulates PD-1 expression, we individually cloned all five genes (VP1-VP5) of IBDV into the pXJ40-myc vector and then transfected them into DT-40 cells to detect PD-1 expression. Western blotting analyses revealed that PD-1 protein expression was significantly upregulated in the VP2 overexpression group compared to that in the other IBDV viral protein overexpression groups (**Figure 2A**). To provide additional evidence, the pXJ40-myc-VP2 plasmid was transfected into DT-40 cells for 24 and 48 h. VP2 overexpression strongly enhanced the protein expression of PD-1 (**Figure 2B**). These data indicate that VP2 upregulates PD-1 protein expression in DT-40 cells.

### 3.3 PD-1 interacts with VP2

**Figure 3:**
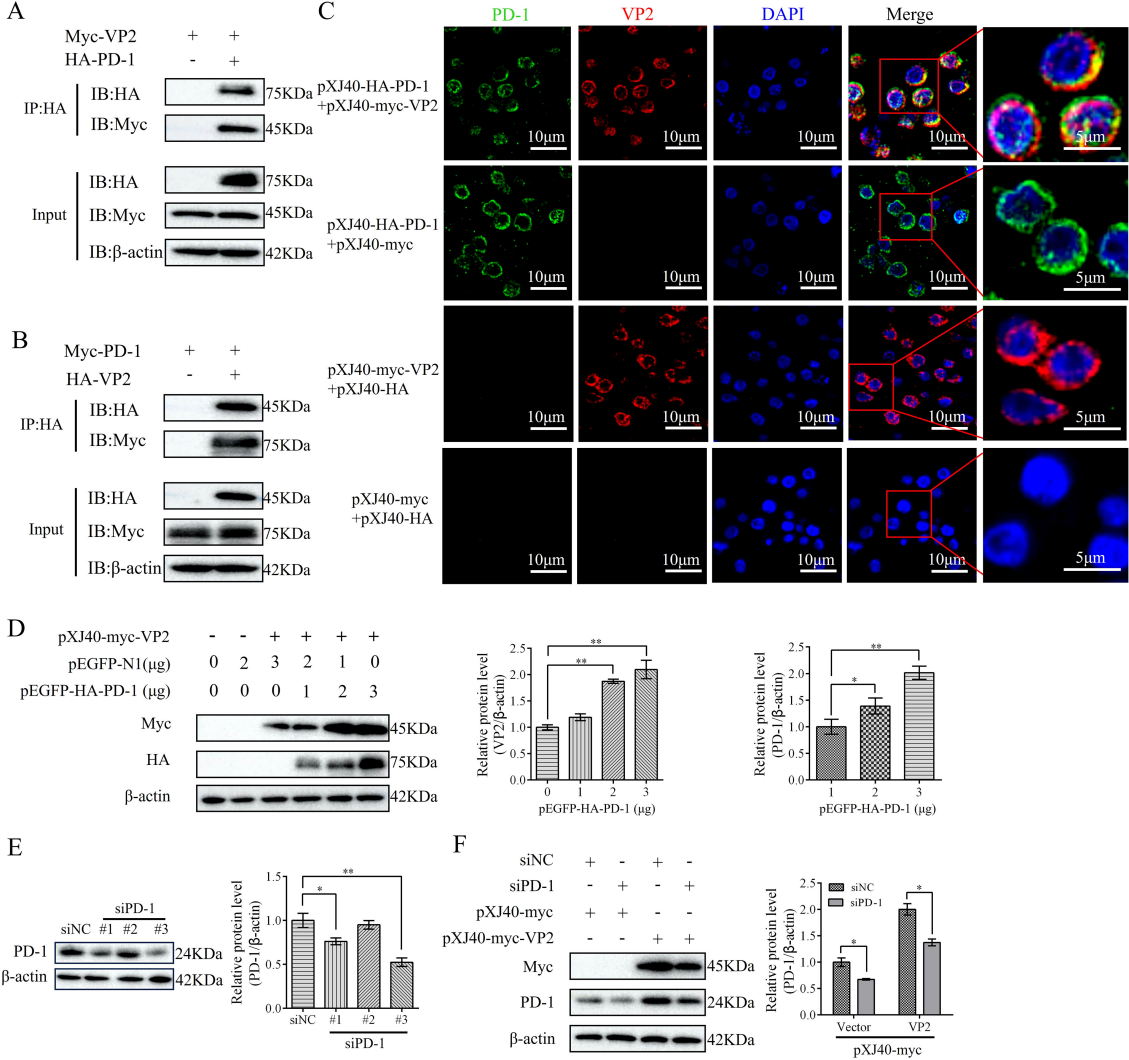
PD-1 interacts with IBDV VP2. **(A)** DT-40 cells were co-transfected with pXJ40-myc-VP2 and pEGFP-HA-PD-1 for 24 h, followed by Co-IP assay. **(B)** DT-40 cells were co-transfected with pXJ40-myc-PD-1 and pEGFP-HA-VP2 for 24 h, followed by Co-IP assay. **(C)** DT-40 cells were co-transfected with pXJ40-HA-PD-1 and pXJ40-myc-VP2 for 24 h, followed by immunofluorescence assay (green, PD-1; red, VP2; blue, nucleus). Scale bar: 10 μm. **(D)** DT-40 cells were co-transfected with different doses of pEGFP-HA-PD-1 plasmids and pXJ40-myc-VP2 for 24 h, followed using western blotting. **(E)** SiRNA knockdown of PD-1 in DT-40 cells transfected with siNC or siPD-1 (#1, #2, and #3), followed by western blotting. **(F)** DT-40 cells were co-transfected with siPD-1 or siNC and pXJ40-myc-VP2 or pXJ40-myc for 24 h, followed using western blotting. Data represented mean ± SD from three replicates. *P < 0.05 and **P < 0.01.

To identify whether PD-1 interacts with VP2, we co-transfected Myc-tagged VP2 and HA-tagged PD-1 or Myc-tagged PD-1 and HA-tagged VP2 into DT-40 cells for 36 h. Co-IP assay results showed that VP2 interacted with PD-1 (**Figures 3A, B**). Further, confocal microscopy revealed that PD-1 colocalized with VP2, which was observed in the same cytoplasmic distribution (**Figure 3C**). Next, We investigated whether PD-1 and VP2 interact directly. Different doses of pEGFP-HA-PD-1 plasmids (0, 1, 2, or 3 µg, respectively) and pXJ40-myc-VP2 were transfected into DT-40 cells for 24 h, and the Myc/HA tag expression was then detected using western blotting. Overexpression of PD-1 was found to enhance VP2 protein expression in a dose-dependent manner (**Figure 3D**). Next. the knockdown efficiency of siPD-1 was confirmed using western blotting (**Figure 3E**). PD-1 silencing inhibited VP2 protein expression in DT-40 cells (**Figure 3F**). These results suggest that PD-1 interacts directly with VP2.

### 3.4 PD-1 promoted IBDV proliferation

**Figure 4:**
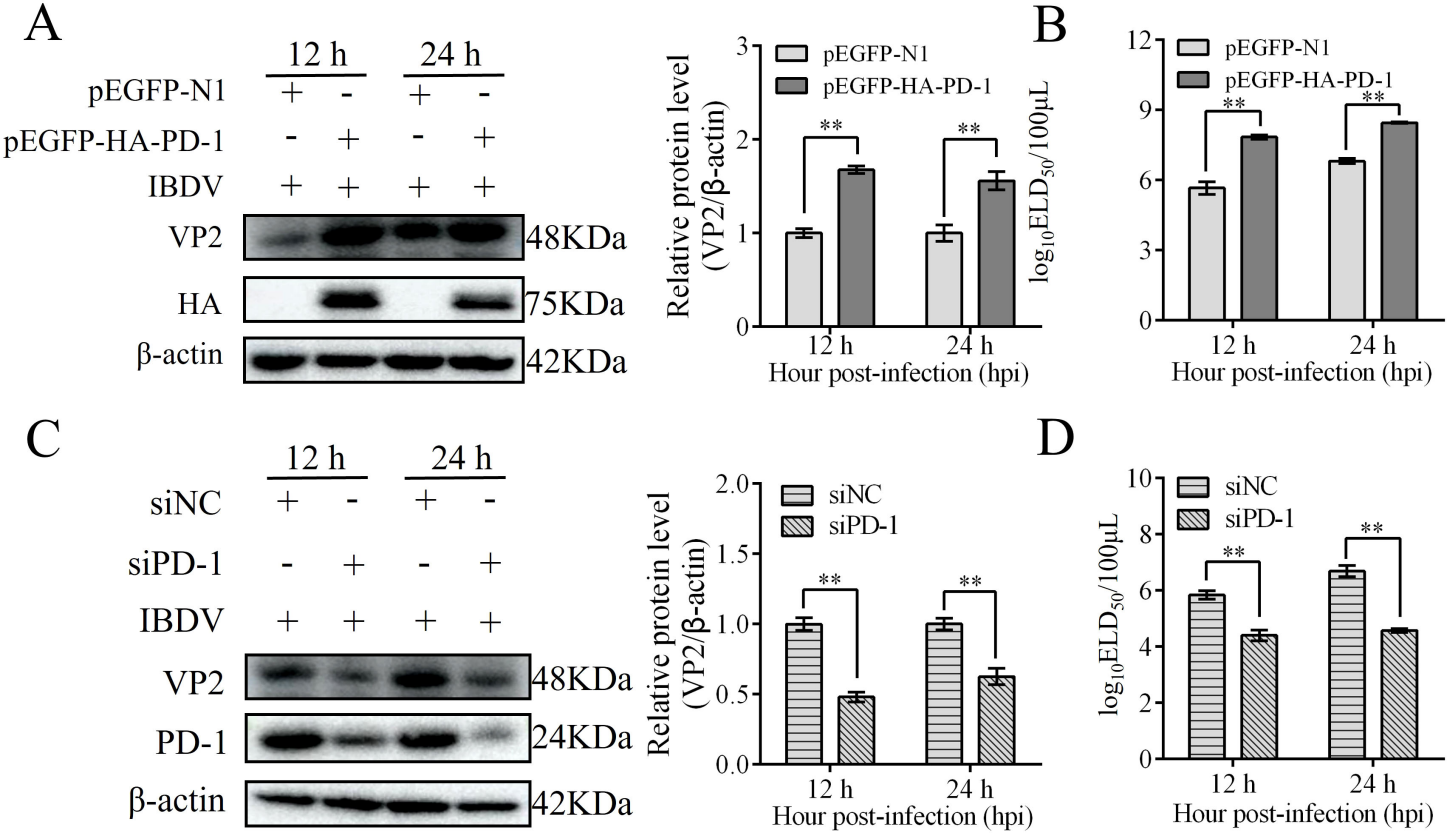
PD-1 promotes IBDV replication. **(A)** DT-40 cells were transfected with pEGFP-N1 or pEGFP-HA-PD-1 for 24 h and then infected them with MOI 1 of IBDV for 12 and 24 h, followed using western blotting. **(B)** DT-40 cells were processed as panel **A**, IBDV titers of extracellular virion production were determined using a 50% egg lethal dose (ELD_50_) assay. **(C)** DT-40 cells were transfected with siNC or siPD-1 for 24 h and then infected them with MOI 1 of IBDV for 12 and 24 h, followed using western blotting. **(D)** DT-40 cells were processed as panel **C**, IBDV titers of extracellular virion production were determined using an ELD_50_ assay. Data represented mean ± SD from three replicates. **P < 0.01.

To elucidate the role of PD-1 in IBDV replication, DT-40 cells were transfected with pEGFP-HA-PD-1 or siPD-1, and then infected with IBDV. Subsequently, western blotting and ELD_50_ assays were used to detect VP2 and IBDV protein expression. Overexpression of PD-1 greatly increased VP2 protein expression and the viral titers compared to that in the control group at 12 and 24 hpi (**Figures 4A, B**). In contrast, silencing PD-1 in DT-40 cells decreased VP2 expression and the IBDV titer (**Figures 4C, D**). These results demonstrate that PD-1 significantly enhances IBDV replication in DT-40 cells.

### 3.5 PD-1 induces autophagy

**Figure 5:**
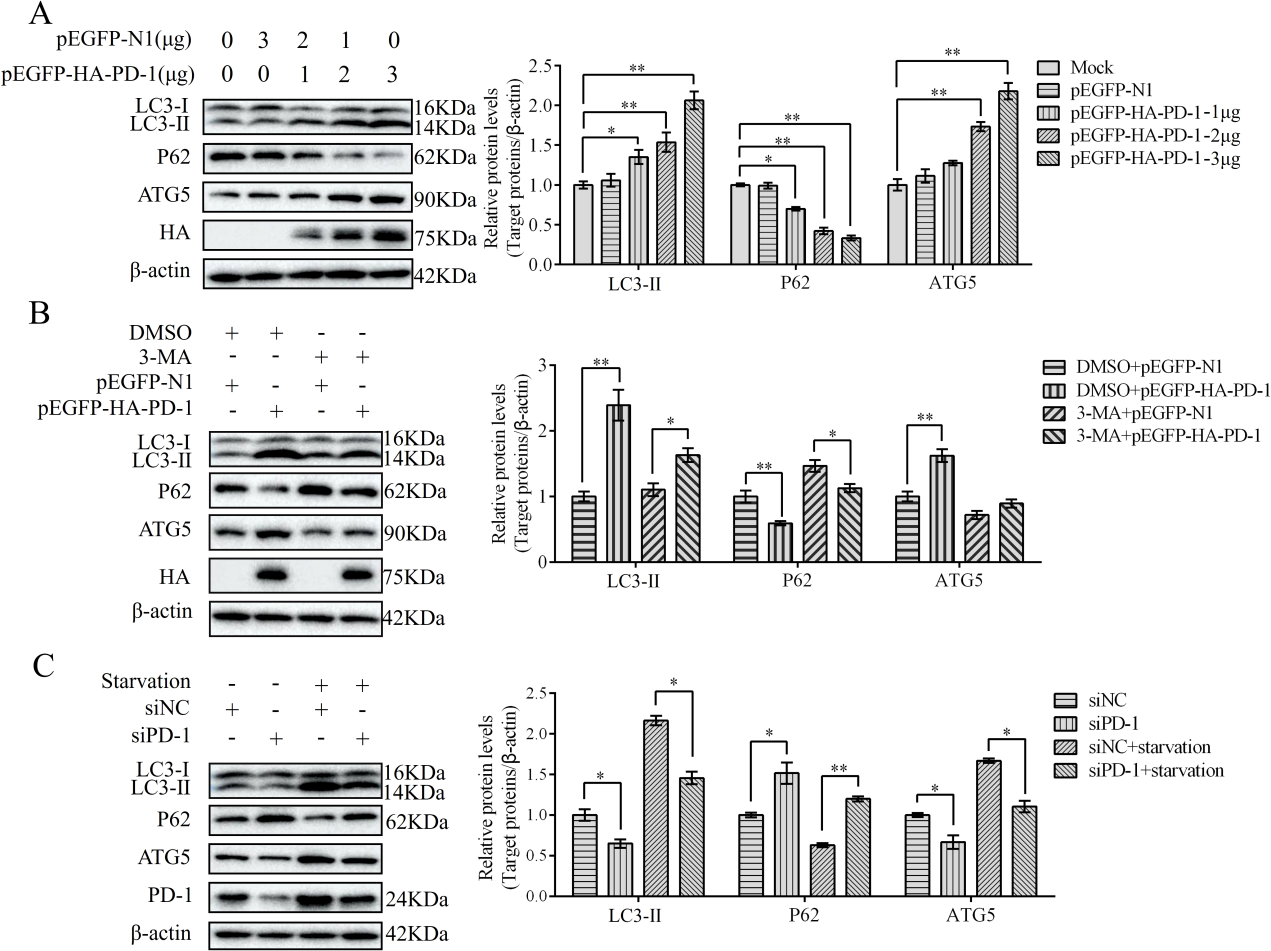
PD-1 induces autophagy. **(A)** DT-40 cells were transfected with different doses of pEGFP-HA-PD-1 plasmids for 24 h, followed by western blotting. **(B)** DT-40 cells were pretreated with dimethyl sulfoxide (DMSO) or 5 mM 3-MA for 6 h and then transfected them with pEGFP-N1 or pEGFP-HA-PD-1 for 24 h, followed using western blotting. **(C)** DT-40 cells were pretreated via starvation with DMEM for 6 h and then transfected them with siNC or siPD-1 for 24 h, followed by western blotting. Data represented mean ± SD from three replicates. *P < 0.05 and **P < 0.01.

To study the characteristics of PD-1-induced autophagy, we examined the expression of autophagy-related proteins (LC3, SQSTM1/p62, and ATG5) in PD-1-transfected DT-40 cells by western blotting. PD-1 overexpression significantly increased the formation of LC3-II, increased the protein levels of ATG5, and decreased the protein level of P62 in a dose-dependent manner (**Figure 5A**). After starvation (a traditional autophagy inducer) or treatment with the autophagy inhibitor 3-MA, autophagy marker proteins were examined. Western blotting results demonstrated that 3-MA treatment decreased the induction of autophagy by PD-1 overexpression, whereas starvation treatment enhanced the inhibition of autophagy induced by silencing PD-1 (**Figures 5B, C**). Overall, these results demonstrate that PD-1 induces autophagy in DT-40 cells.

### 3.6 IBDV induces autophagy

**Figure 6:**
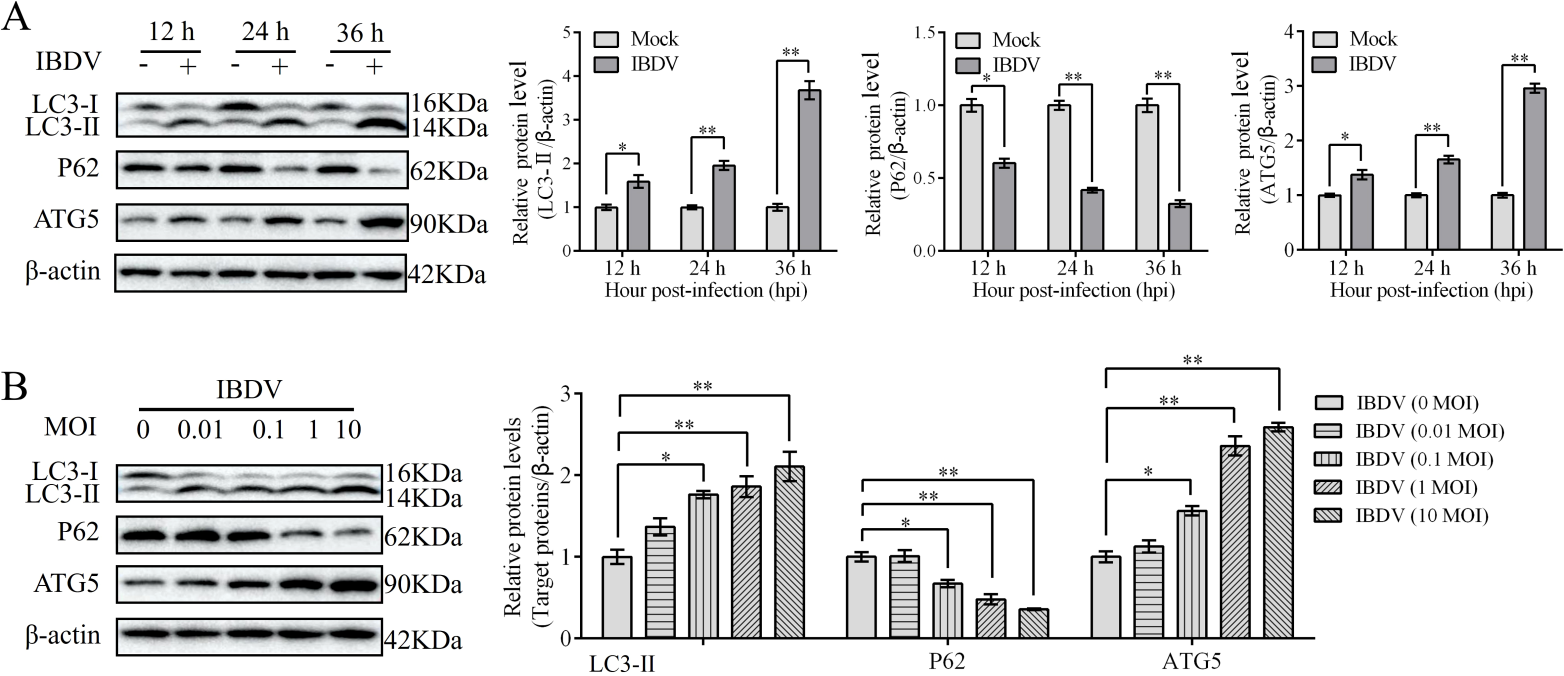
IBDV induces autophagy. **(A)** DT-40 cells were infected with MOI 1 of IBDV for 12, 24, and 36 h, followed using western blotting. **(B)** DT-40 cells were infected with or without IBDV at different MOIs for 24 h, followed using western blotting. Data represented mean ± SD from three replicates. *P < 0.05 and **P < 0.01.

To further confirm IBDV-induced autophagy, DT-40 cells were infected with IBDV for 12, 24, and 36 h, and the expression of autophagic marker proteins was examined by western blotting. IBDV infection significantly increased LC3-II puncta accumulation and ATG5 expression but attenuated the protein expression of P62 compared to those in the mock group at 24 and 36 h (**Figure 6A**). Next, DT-40 cells were infected with IBDV at different MOI (0, 0.01, 0.1, 1, and 10). We observed a remarkable dose-dependent relationship between IBDV infection and the autophagy-related proteins (**Figure 6B**). Overall, these results imply that IBDV infection induces autophagy in DT-40 cells.

### 3.7 PD-1 promotes autophagy induced by IBDV

**Figure 7:**
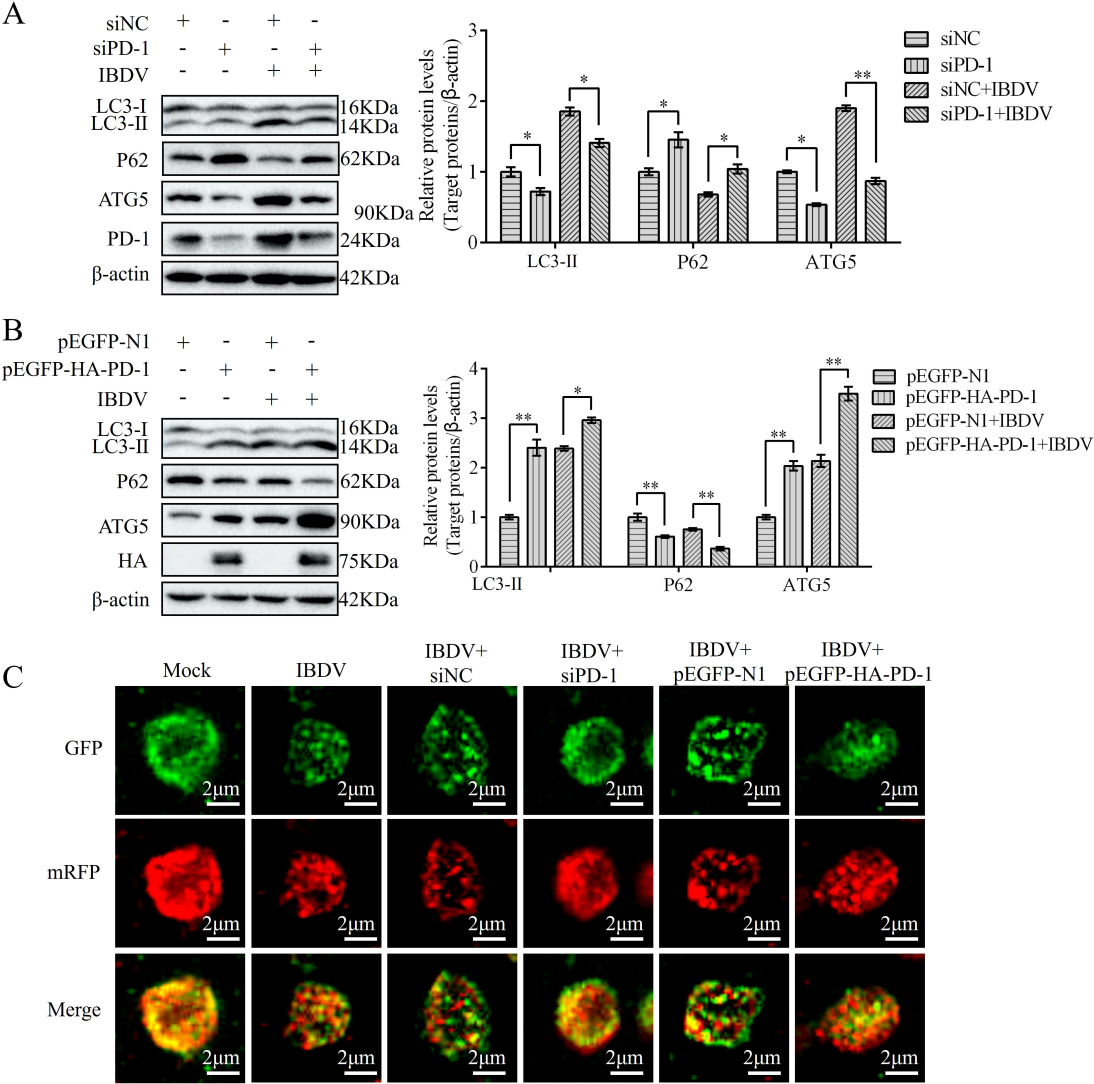
PD-1 promotes autophagy induced by IBDV. **(A)** DT-40 cells were transfected with siNC or siPD-1 for 24 h and then infected them with MOI 1 of IBDV for 24 h, followed using western blotting. **(B)** DT-40 cells were transfected with pEGFP-N1 or pEGFP-HA-PD-1 for 24 h and then infected with MOI 1 of IBDV for 12 and 24 h, followed using western blotting. **(C)** DT-40 cells were co-transfected with mRFP-GFP-LC3B and siPD-1 or pEGFP-HA-PD-1 for 24 h and then infected them with MOI 1 of IBDV for 24 h, the laser confocal microscope was used to observe the expressions of fluorescence (red and green) in DT-40 cells. Scale bar: 2 μm. Data represented mean ± SD from three replicates. *P < 0.05 and **P < 0.01.

Previous studies have demonstrated that IBDV induces autophagy, which promotes replication (21). To confirm whether PD-1 is involved in IBDV-induced autophagy, we transfected DT-40 cells with siPD-1 or pEGFP-HA-PD-1 and then infected them with IBDV to analyze the proteins associated with autophagy markers. Western blotting analyses revealed that PD-1 knockdown reduced the protein expression of LC3-II and ATG5and increased P62 expression in DT-40 cells, whereas IBDV infection reduced the inhibition of autophagy in PD-1 knockdown DT-40 cells (**Figure 7A**). In contrast, PD-1 overexpression significantly enhanced LC3-II and ATG5 expression, while decreasing the protein expression of P62 in DT-40 cells (**Figure 7B**). Meanwhile, IBDV infection enhanced the autophagy induced by PD-1 overexpression. Next, mRFP-GFP-LC3 was introduced into DT-40 cells to analyze the autophagy flux. As shown in **Figure 7C**, PD-1 overexpression resulted in augmented red fluorescence caused by IBDV infection, whereas silencing PD-1 resulted in the opposite effect in IBDV-infected DT-40 cells. These results suggest that PD-1 promotes IBDV-induced autophagy.

### 3.8 PD-1 inhibits the PI3K/AKT pathway in IBDV infection

**Figure 8:**
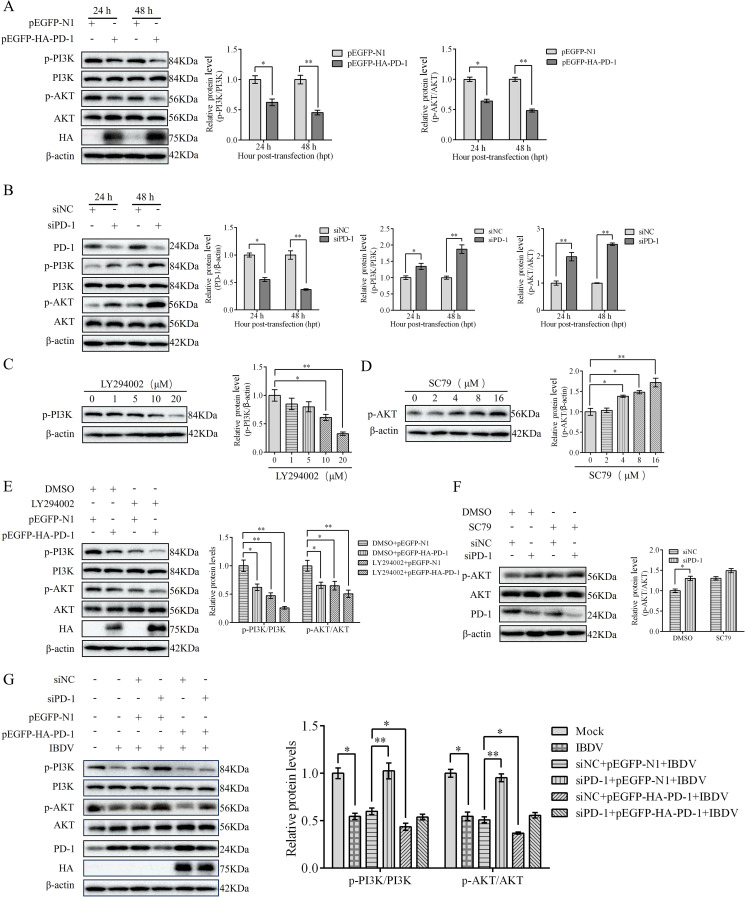
PD-1 inhibits PI3K/AKT pathway in IBDV infection. **(A)** DT-40 cells were transfected with pEGFP-N1 or pEGFP-HA-PD-1 plasmids for 24 and 48 h, followed by western blotting. **(B)** DT-40 cells were transfected with siNC or siPD-1 for 24 and 48 h, followed using western blotting. **(C)** DT-40 cells were treated with different doses of LY294002 for 12 h, followed using western blotting. **(D)** DT-40 cells were treated with different doses of SC79 for 6 h, followed using western blotting. **(E)** DT-40 cells were pretreated with DMSO or 10 μM LY294002 for 2 h and then transfected them with pEGFP-HA-PD-1 for 24 h, followed using western blotting. **(F)** DT-40 cells were pretreated with DMSO or 8 μM SC79 for 2 h and then transfected with siNC or siPD-1 for 24 h, followed using western blotting. **(G)** DT-40 cells were transfected with pEGFP-HA-PD-1 and/or siPD-1 for 24 h and then infected them with MOI 1 of IBDV for 24 h, followed using western blotting. Data represented mean ± SD from three replicates. *P < 0.05 and **P < 0.01.

We have previously reported that the PI3K/AKT pathway is inactivated in IBDV-infected DT-40 cells (14). To elucidate the mechanism by which PD-1-induces autophagy, we first examined the effect of PD-1 on the PI3K/AKT signaling pathway. Western blotting analysis showed that PD-1 overexpression strongly reduced the protein expression of p-PI3K, whereas PD-1 silencing resulted in the opposite effect (**Figures 8A, B**). Next, we observed the effects of the PI3K/AKT pathway inhibitor LY294002 and the activator SC79 on PI3K and AKT in DT-40 cells. LY294002 significantly suppressed p-PI3K expression in a dose-dependent manner, whereas SC79 greatly enhanced p-AKT expression in a dose-dependent manner (**Figures 8C, D**). Moreover, LY294002 strongly increased the inhibitory effect of PD-1 on p-PI3K, whereas SC79 strongly reduced the inhibition of p-AKT induced by PD-1 knockdown (**Figure 8E, F**). As expected, PD-1 overexpression decreased the protein expression of p-PI3K and p-AKT in IBDV-infected DT-40 cells compared to that in uninfected mock cells, while silencing of PD-1 attenuated the activation of the PI3K/AKT pathway (**Figure 8G**). These results revealed that PD-1 suppresses the PI3K/AKT pathway during IBDV infection.

### 3.9 PD-1 promotes the activation of FoxO1 protein in IBDV infection

**Figure 9:**
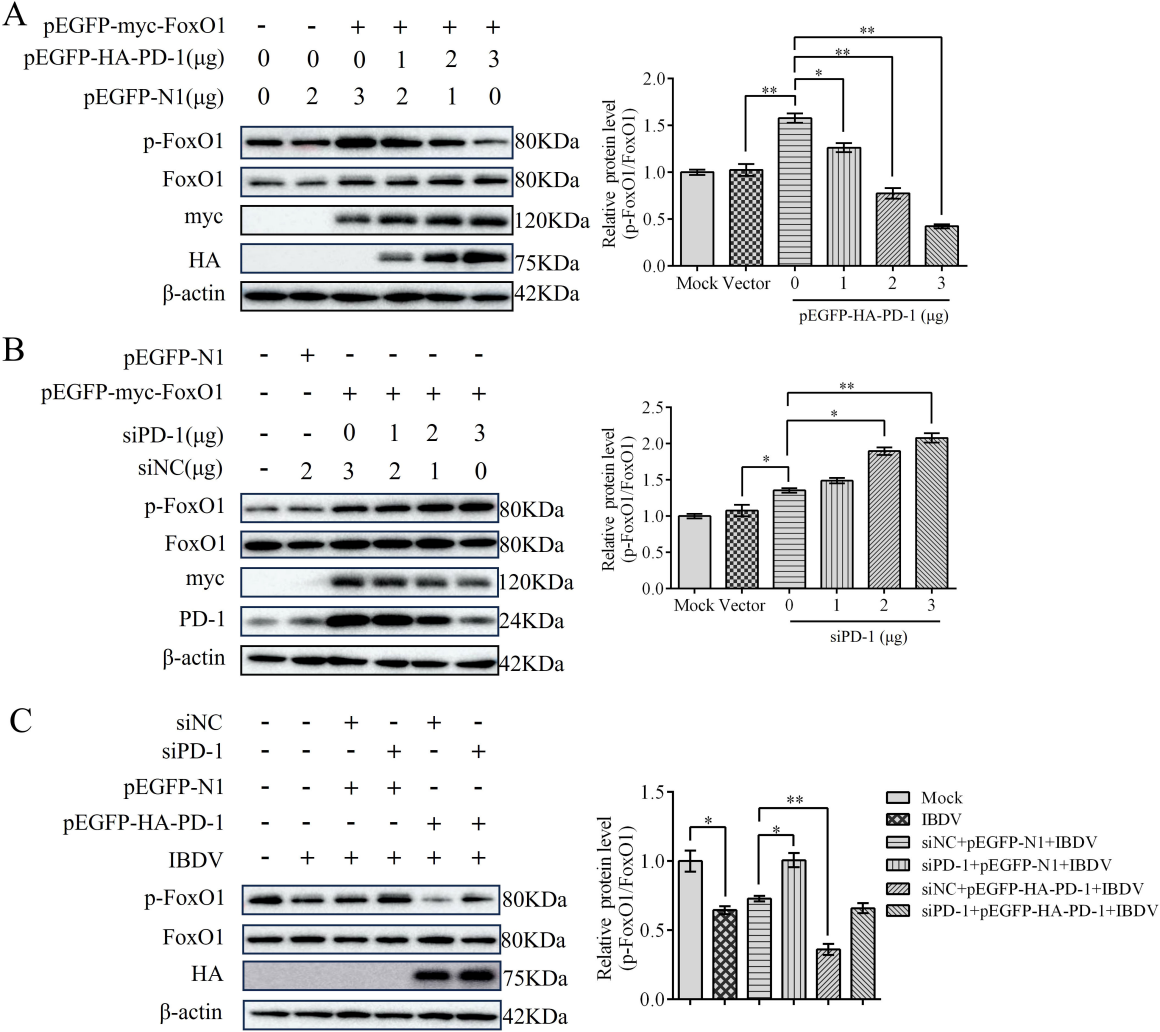
PD-1 contributes to the activation of FoxO1 protein in IBDV infection. **(A)** DT-40 cells were co-transfected with pEGFP-myc-FoxO1 and different doses of pEGFP-HA-PD-1 plasmids for 24 h, followed using western blotting. **(B)** DT-40 cells were co-transfected with pEGFP-myc-FoxO1 and different doses of siPD-1 for 24 h, and then infected them with MOI 1 of IBDV for 12 h, followed using western blotting. **(C)** DT-40 cells were transfected with pEGFP-myc-FoxO1 and/or siPD-1 for 24 h, and then infected with MOI 1 of IBDV for 12 h, followed using western blotting. Data represented mean ± SD from three replicates. *P < 0.05 and **P < 0.01.

To explore whether FoxO1 was triggered by PD-1, DT-40 cells were co-transfected with pEGFP-HA-PD-1 and pEGFP-myc-FoxO1 plasmids for 24 h. We found that p-FoxO1 protein expression was decreased in PD-1 overexpressed DT-40 cells in a dose-dependent manner, whereas siPD-1 enhanced the protein expression of p-FoxO1 in FoxO1 overexpressed DT-40 cells (**Figures 9A, B**). As shown in **Figure 9C**, siPD-1 greatly enhanced the protein expression of p-FoxO1 in IBDV-infected DT-40 cells, whereas PD-1 overexpression greatly reduced the protein expression of p-FoxO1. These results suggest that overexpression of PD-1 significantly increases FoxO1 activation in IBDV infection, whereas PD-1 knockdown shows a contrasting effect.

### 3.10 PD-1 promotes FoxO1 activity by inhibiting PI3K/AKT pathway

**Figure 10:**
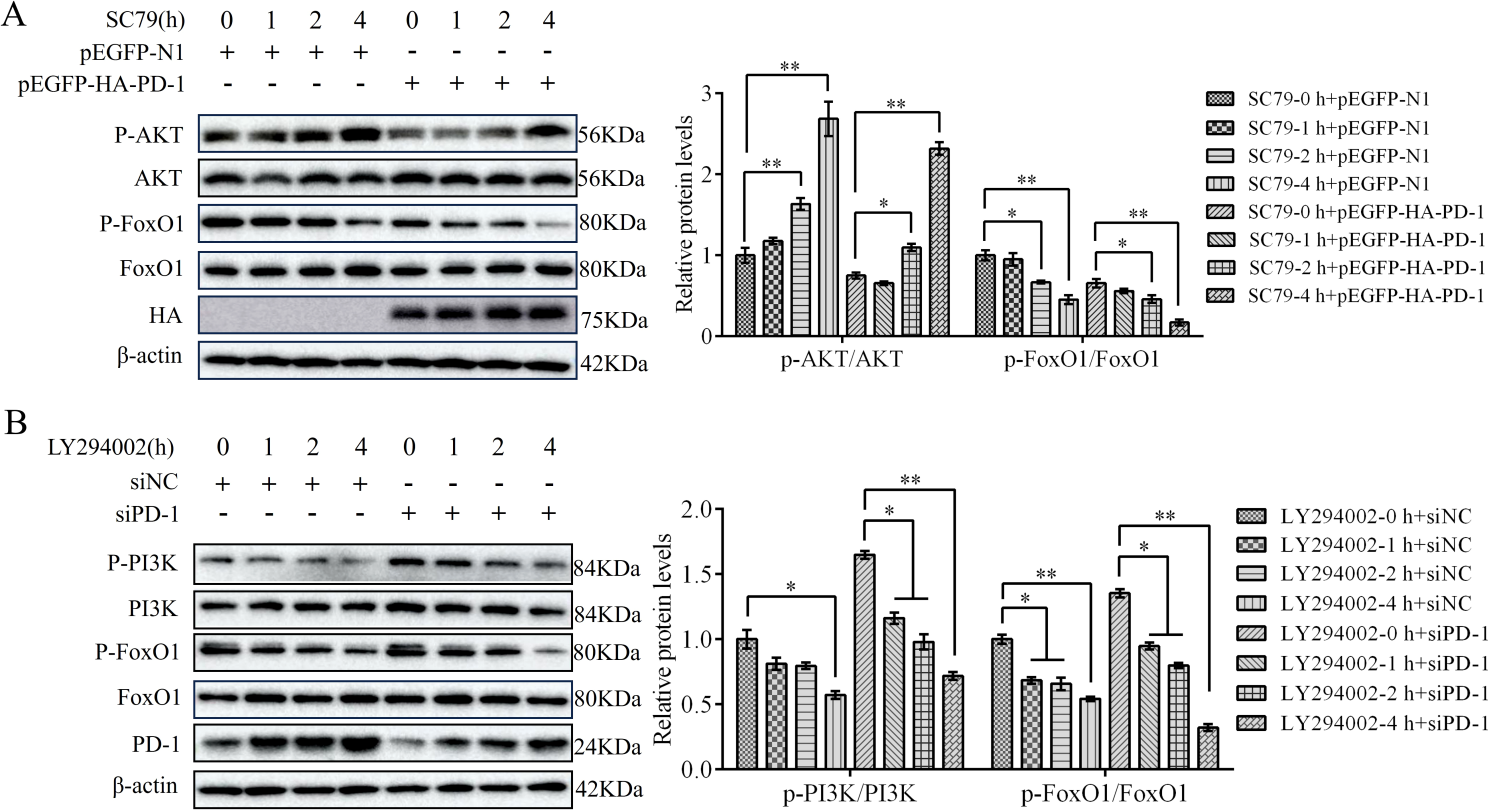
PD-1 promotes FoxO1 activity by inhibiting PI3K/AKT pathway. **(A)** DT-40 cells were pretreated with DMSO or SC79 for 0, 1, 2, and 4 h and then transfected them with pEGFP-N1 or pEGFP-HA-PD-1 plasmids for 24 h, followed using western blotting. **(B)** DT-40 cells were pretreated with DMSO or 10 μM LY294002 for 0, 1, 2, and 4 h and then transfected them with siNC or siPD-1 for 24 h, followed using western blotting with an anti-PD-1, anti-p-AKT, anti-AKT, anti-p-FoxO1, and anti-FoxO1 antibodies. Data represented mean ± SD from three replicates. *P < 0.05 and **P < 0.01.

Next, we investigated the effect of the PI3K/AKT pathway on PD-induced FoxO1 activity. To this end, we transfected PD-1 plasmids or siPD-1 into DT-40 cells for 24 h after treatment with SC79 or LY294002 for 0, 1, 2, and 4 h. Western blotting analysis showed that PD-1 overexpression greatly reduced the protein expression of p-AKT and p-FoxO1 compared to that in the control group (**Figure 10A**). Conversely, PD-1 silencing resulted in the opposite effect (**Figure 10B**). Furthermore, SC79 treatment significantly reduced the protein expression of p-PI3K and p-FoxO1 in PD-1-overexpressing DT-40 cells, whereas LY294002 attenuated the inhibitory effect of siPD-1 on p-PI3K and p-FoxO1 (**Figures 10A, B**). These results further indicate that PD-1 promotes FoxO1 expression by inhibiting PI3K/AKT in DT-40 cells.

### 3.11 PD-1 induced autophagy through the PI3K/AKT/FoxO1 pathway

**Figure 11:**
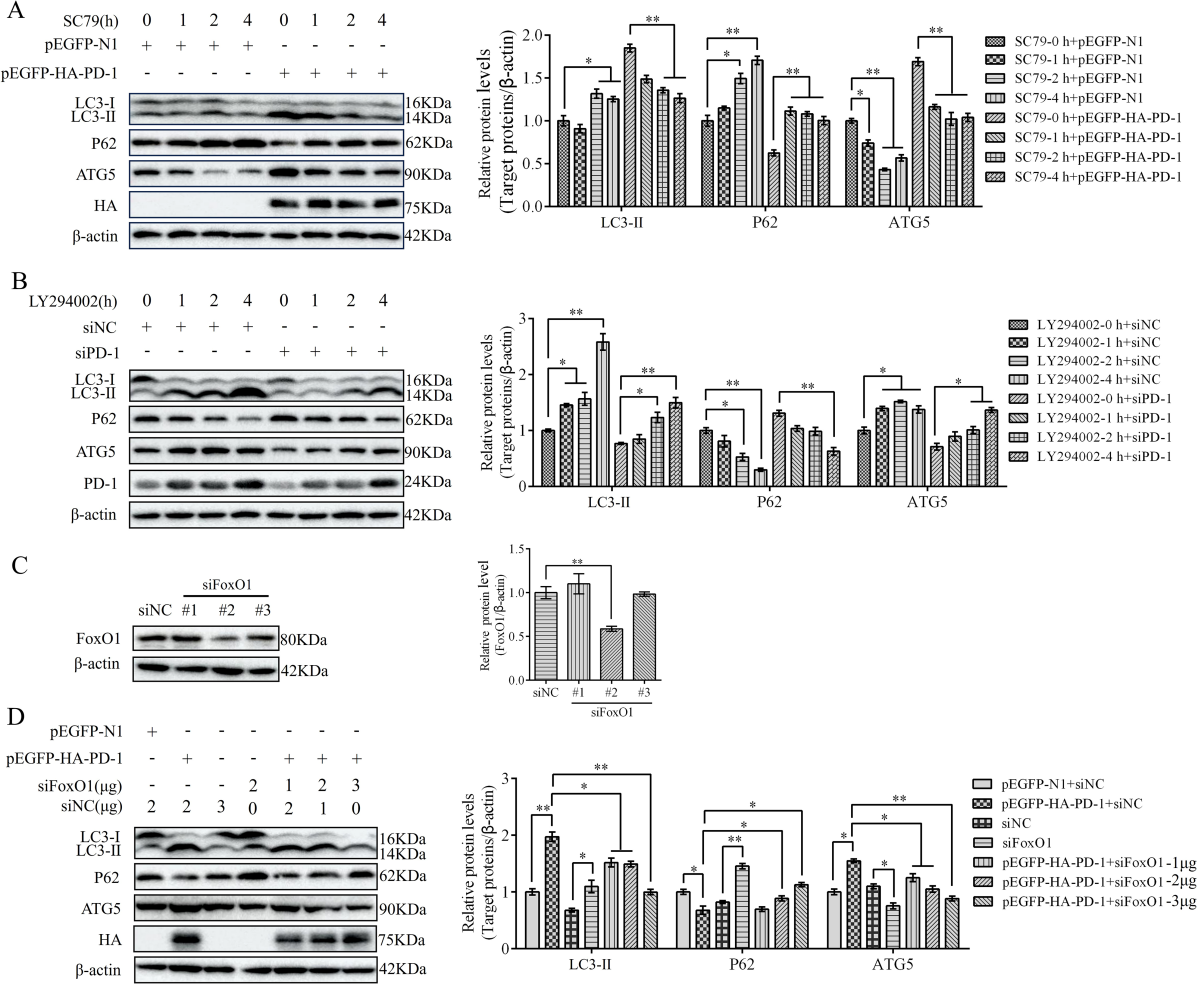
PD-1 induces autophagy by suppressing PI3K/AKT FoxO1 pathway. **(A)** DT-40 cells were pretreated with DMSO or 8 μM SC79 for 0, 1, 2, and 4 h and then transfected them with pEGFP-N1 or pEGFP-HA-PD-1 for 24 h, followed using western blotting. **(B)** DT-40 cells were pretreated with DMSO or 10 μM LY294002 for 0, 1, 2, and 4 h and then transfected them with siNC or siPD-1 for 24 h, followed using western blotting. **(C)** DT-40 cells were transfected with siNC or siFoxO1 (#1, #2, and #3). The knockdown efficiency of FoxO1 was identified by western blotting. **(D)** DT-40 cells were co-transfected with pEGFP-HA-PD-1 and different doses of siPD-1 for 24 h, followed using western blotting. Data represented mean ± SD from three replicates. *P < 0.05 and **P < 0.01.

To investigate whether PD-1 induces autophagy via the activation of PI3K/AKT/FoxO1 pathway, we transfected pEGFP-HA-PD-1 or siPD-1 into DT-40 cells after treatment with SC79 or LY294002 for 0, 1, 2, and 4 h. Western blotting analysis revealed that SC79 treatment restrained the protein expression of LC3 and ATG5 and enhanced the protein expression of P62 in PD-1-overexpressing DT-40 cells, whereas LY294002 attenuated the inhibitory effect of siPD-1 on autophagy (**Figures 11A, B**). Although PD-1 is known to participate in FoxO1 activity, it remains unclear whether FoxO1 is essential for autophagy induction by PD-1 in DT-40 cells. To further verify the promoting effect of FoxO1 on autophagy, we co-transfected pEGFP-HA-PD-1 and siFoxO1 or siPD-1 and pEGFP-myc-FoxO1 into DT-40 cells for 24 h and detected autophagy-related proteins using western blotting analysis. The knockdown efficiency of FoxO1 in DT-40 cells was confirmed (**Figure 11C**). As shown in **Figure 11D**, silencing FoxO1 attenuated the protein expression of LC3-II and ATG5 and promoted the protein expression of P62 in PD-1-overexpressing DT-40 cells in a dose-dependent manner. These results indicated that PD-1 induces autophagy via the PI3K/AKT/FoxO1 pathway.

### 3.12 PD-1 promotes IBDV replication via the PI3K/AKT/FoxO1 pathway

**Figure 12:**
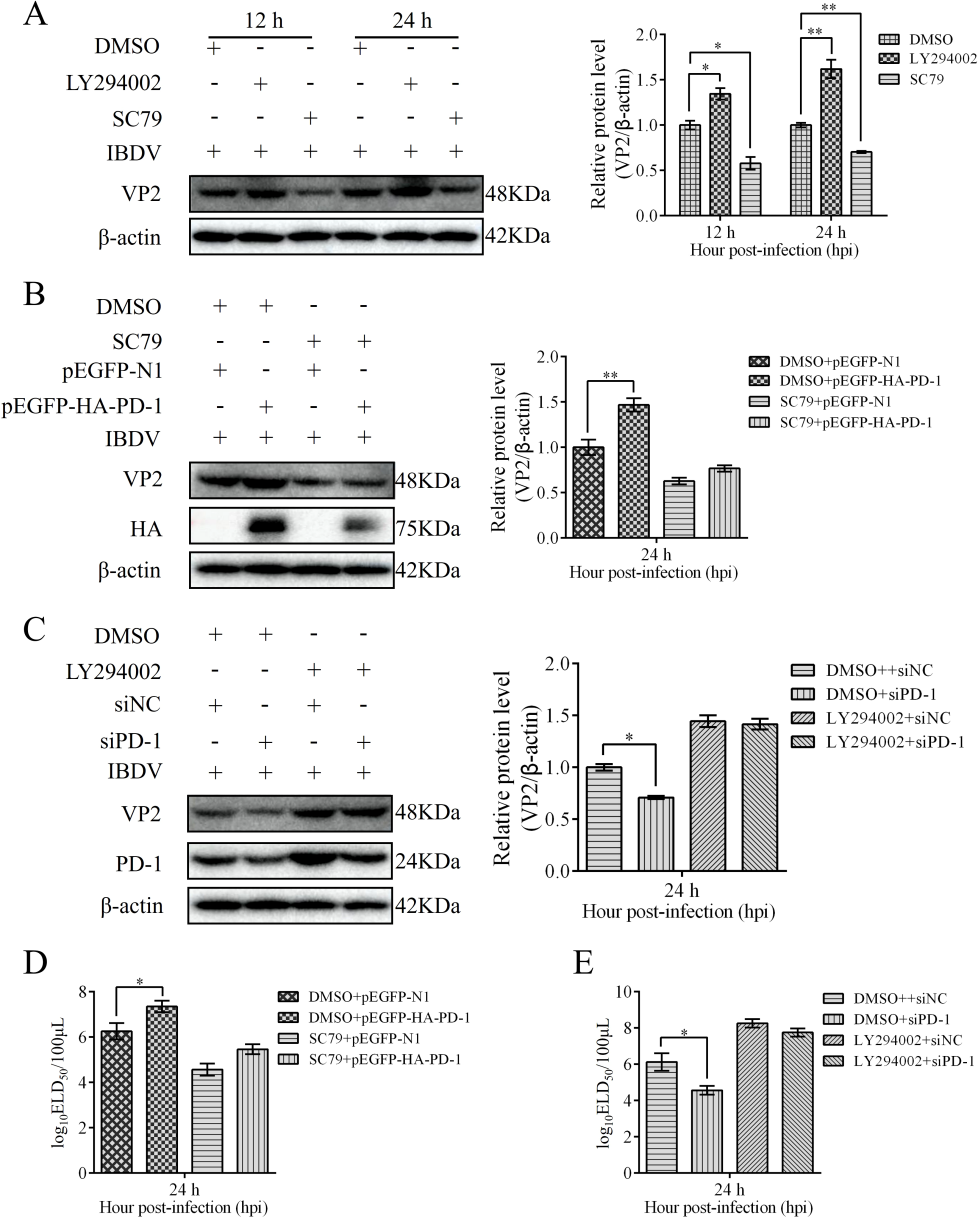
PD-1 promotes IBDV proliferation through the PI3K/AKT/FoxO1 pathway. **(A)** DT-40 cells were pretreated with 10 μM LY294002 or 8 μM SC79 for 2 h and then infected them with MOI 1 of IBDV for 12 and 24 h, followed using western blotting. **(B)** DT-40 cells were pretreated with DMSO or 8 μM SC79 for 2 h and then transfected them with pEGFP-HA-PD-1 and subsequently infected with MOI 1 of IBDV for 36 h, followed using western blotting. **(C)** DT-40 cells were pretreated with DMSO or 10 μM LY294002 for 2 h and then transfected them with siNC or siPD-1 and subsequently infected them with MOI 1 of IBDV for 24 h, followed using western blotting. **(D)** DT-40 cells were processed as panel **(B)** IBDV titers of extracellular virion production were determined using an ELD_50_ assay. **(E)** DT-40 cells were processed as panel **(C)** IBDV titers of extracellular virion production were determined using an ELD_50_ assay. Data represented mean ± SD from three replicates. *P < 0.05 and **P < 0.01.

To evaluate the role of the PI3K/AKT/FoxO1 pathway on IBDV replication, we treated DT-40 cells with LY294002 or SC79 before IBDV infection and analyzed the protein expression of VP2. Western blotting showed that LY294002 treatment obviously enhanced the protein expression of VP2 in IBDV-infected DT-40 cells, whereas SC79 significantly decreased the protein expression of VP2 (**Figure 12A**). To further determine whether PD-1 promotes IBDV replication via the PI3K/AKT pathway, we transfected PD-1 or siPD-1 into DT-40 cells and then infected them with IBDV after treatment with LY294002 or SC79 followed by detection of VP2 protein and IBDV replication using western blotting and ELD50 analysis, respectively. We observed that SC79 treatment significantly reduced the VP2 protein expression level and IBDV titers in PD-1-overexpressing DT-40 cells with IBDV infection compared with those in the control cells infected with IBDV (**Figures 12B, D**), whereas LY294002 treatment significantly enhanced the protein expression of VP2 and the titers of IBDV in PD-1-knockdown DT-40 cells with IBDV infection (**Figure 12C, E**). Overall, these results implied that PD-1 promoted IBDV proliferation by activating the PI3K/AKT/FoxO1 pathway.

### 3.13 PD-1 promotes IBDV replication via autophagy

**Figure 13:**
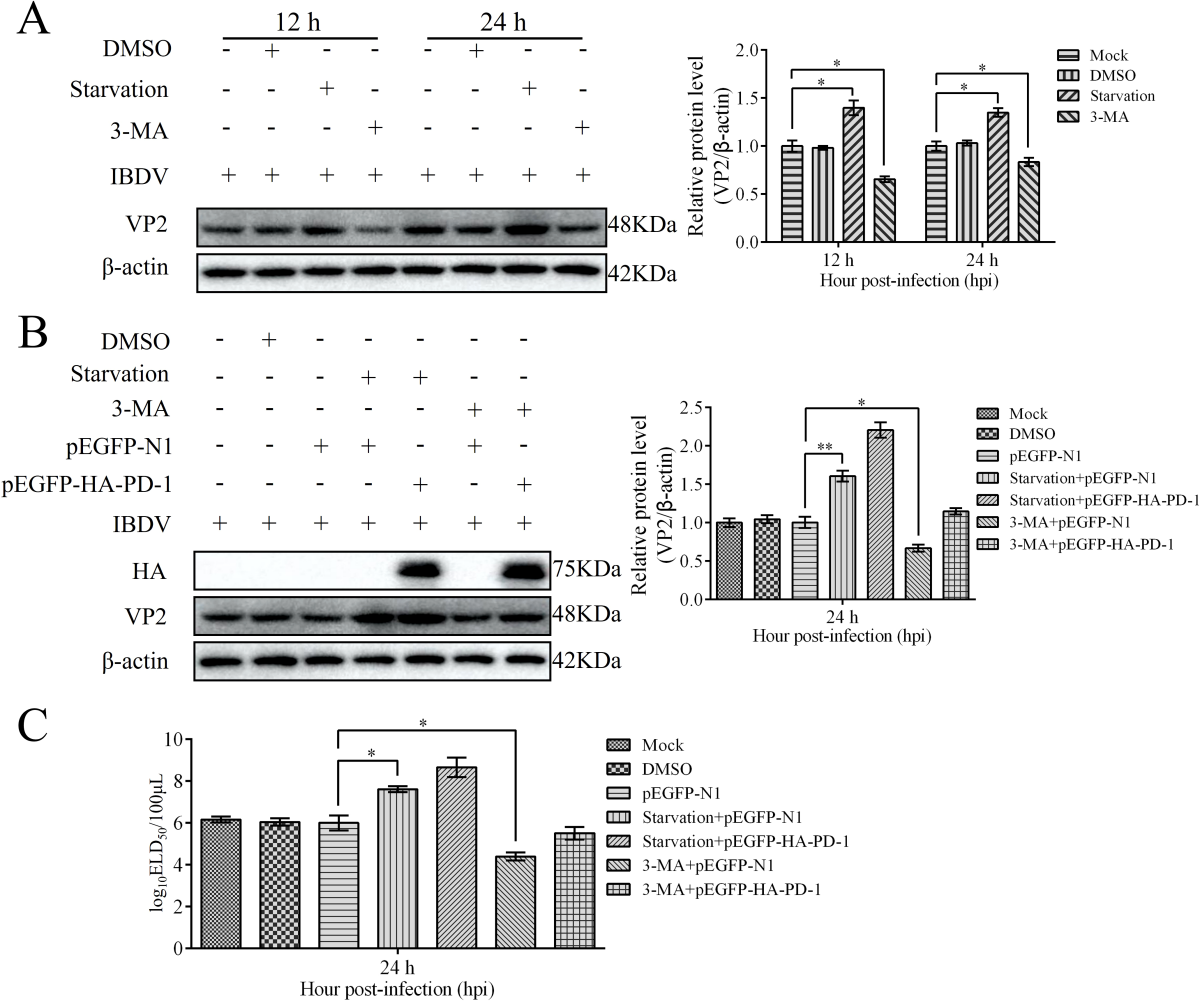
PD-1 overexpression promotes IBDV proliferation via autophagy. **(A)** DT-40 cells were pretreated via starvation with DMEM for 6 h or complete medium containing 5 mM 3-MA for 6 h and then infected them with MOI 1 of IBDV for 24 h, followed using western blotting. **(B)** DT-40 cells were transfected with pEGFP-N1 or pEGFP-HA-PD-1 for 24 h and then infected them with IBDV at an MOI of 1 for 24 h after starvation for 6 h or treatment with 5 mM 3-MA for 6 h, followed using western blotting. **(C)** DT-40 cells were processed as panel **B**, IBDV titers of extracellular virion production were determined using an ELD_50_ assay. Data represented mean ± SD from three replicates. *P < 0.05 and **P < 0.01.

To determine the effect of autophagy on IBDV replication, we infected DT-40 cells with IBDV after starvation or treatment with the autophagy inhibitor 3-MA. Western blotting analyses showed that starvation greatly enhanced the protein expression of VP2 compared with that in the control group, whereas 3-MA significantly decreased the protein expression of VP2. Next, we examined the relationship between PD-1-induced autophagy and IBDV replication (**Figure 13A**). After starvation or treatment with the autophagy inhibitor 3-MA, DT-40 cells were transfected with pEGFP-HA-PD-1 and then infected with IBDV at an MOI of 1. We observed that starvation increased VP2 protein expression and the IBDV titers in IBDV-infected DT-40 cells (**Figures 13B, C**). Further, VP2 protein levels and IBDV titers were significantly increased in PD-1 overexpressing DT-40 cells infected with IBDV after starvation, whereas 3-MA treatment resulted in the opposite effect (**Figures 13B, C**). These results suggest that PD-1-induced autophagy is beneficial for IBDV replication.

## 4 Discussion

**Figure 14:**
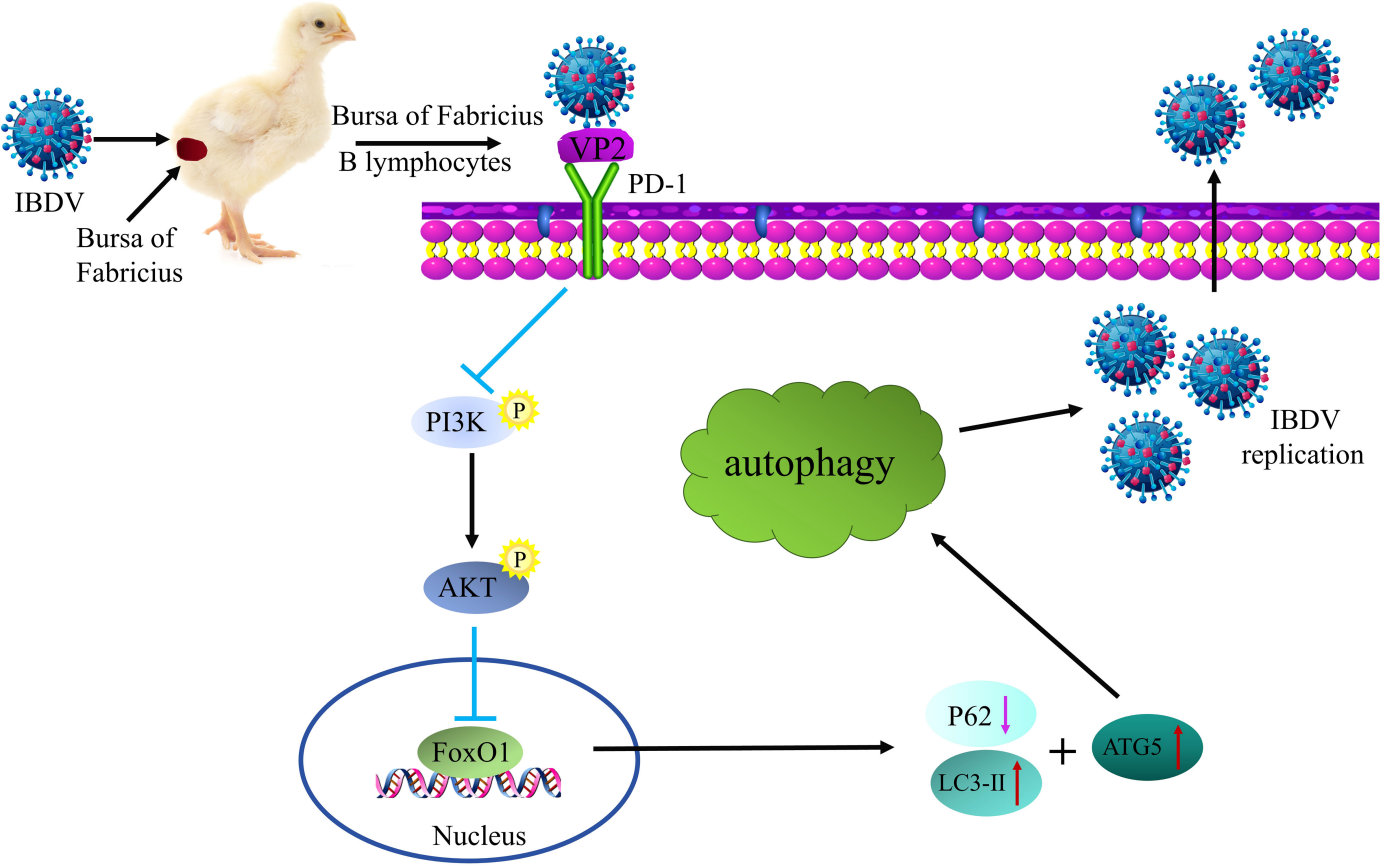
Schematic diagram of IBDV utilizing PD-1-induced autophagy to promote viral replication. Upon IBDV infection, PD-1 protein expression is significantly upregulated in the BF of chickens and B lymphocytes. Furthermore, PD-l directly interacts with viral protein VP2 to enhance the induction of autophagy through the PI3K/AKT-FoxOl axis, which contributes to virus replication and release.

IBDV replication is strictly regulated by host factors during infection. Studies have shown that the relationship between viruses and their host factors significantly influences the pathogenesis of multiple viral diseases such as hepatitis B virus, bovine viral diarrhea virus, and African swine fever virus (ASFV) (31–33). Therefore, exploring the infection strategies of viruses in host cells can help further analyze the pathogenesis of IBDV, which can contribute to the development of more effective antiviral therapeutics to prevent IBDV infection. In this study, we found that IBDV infection significantly upregulated PD-1 expression, which induced autophagy via the PI3K/AKT/FoxO1 pathway for viral replication (**Figure 14**). Thus, our findings reveal a new direction for deeper understanding of the immune system mechanisms and pathogenesis of IBDV.

PD-1 plays a unique regulatory role in controlling viral infections and contributes to the immune evasion mechanism that causes B-lymphocyte dysfunction (34, 35). Studies have shown that B-cell activation via immune-mediated stimulation is the main method for initiating viral protein synthesis (27). Blocking bovine PD-1 inhibits bovine leukemia virus replication in B cells (12). Similarly, our previous study indicated that blocking the PD-1/PD-L1 interaction restores B lymphocyte function during IBDV infection (14). Here, we found that PD-1 was greatly enhanced by IBDV infection in the BF of chickens and DT-40 cells, and was required for IBDV replication.

Viruses rely on host cell components for adapting to unfavorable host environments for efficient replication. For example, porcine epidemic diarrhea virus N protein interacts with and enhances TRIM28 in host cells, which promotes viral release (36). Similarly, the ASFV CD2v protein interacts with host CSF2RA to facilitate ASFV replication (37). Growing evidence suggests that IBDV can interact with some membrane proteins such as surface IgM, HSP90AA1, and HSC70, all of which support viral particle release (38–40). The IBDV VP2 capsid protein is recognized as a medium between the host factors and virus invasion (41). Previous studies have indicated that CD44 and/or CD74, as cellular receptors, interact with the viral protein VP2, which facilitates IBDV infection in DT-40 cells (30, 42). In our study, we found that the IBDV structural protein VP2 interacts with PD-1 to facilitate viral replication. Additionally, PD-1 overexpression promoted IBDV replication, whereas PD-1 knockdown inhibited IBDV replication.

Autophagy is an intracellular degradation system that maintains normal cellular function by breaking down damaged organelles and misfolded or aggregated proteins, thereby facilitating the clearance of harmful substances from the cells (43). However, autophagy potentially exerting antiviral functions or being utilized by viruses to promote their own replication and transmission. Previous studies have indicated that some viruses utilize autophagic mechanisms to promote their replication, whereas others are suppressed by autophagy (44). Notably, some RNA viruses can inhibit or even utilize host cell autophagy to enhance their propagation. For instance, Newcastle disease virus induces autophagy in DF-1 cells to promote its pathogenicity (19). Although some studies have demonstrated that autophagy is triggered by RNA viruses, its role in RNA viral replication remains to be fully elucidated. As one of the important model systems for RNA viruses, we revealed that IBDV infection induces autophagic lysosome formation in DT-40 cells. Additionally, PD-1 overexpression promotes autophagy induction, whereas PD-1 silencing inhibits autophagy induction. However, induction of autophagy by PD-1 during IBDV infection remains largely unknown.

The PI3K/AKT pathway negatively regulates autophagy and promotes viral replication during infection. For example, canine distemper virus induces cellular autophagy by suppressing the PI3K/AKT pathway to facilitate its replication and propagation (45). Similarly, ZIKV infection promotes autophagy via the PI3K/AKT pathway to enhance its own replication (46). Additionally, we recently reported that the PI3K/AKT signaling pathway was suppressed after IBDV infection (14). PD-1 has been shown to inhibit BCR signaling and negatively regulate B lymphocyte function in immunosuppressive diseases (13). Furthermore, PD-1 blockade promotes BCR signaling and activates the PI3K/AKT pathway in cancer cells (13, 47). Although PI3K/AKT pathway activation by BCR signaling is well understood, the specific mechanism of the PI3K/AKT pathway in host cells infected with IBDV is not well defined. Our study first examined the relationship between PD-1 and the PI3K/AKT pathway during IBDV infection and revealed that PD-1 overexpression suppressed the PI3K/AKT pathway, and that IBDV infection decreased the inhibitory effect of siPD-1 on the PI3K/AKT pathway. One study revealed that autophagy induction triggered by IBDV infection remarkably inhibited the AKT-mTOR pathway (48). However, further studies are needed to elucidate the detailed mechanism about the network of PD-1-induced autophagy and PI3K/AKT pathway in pathogenesis of IBDV.

FoxO1 is well-known as a major transcription factor downstream of the PI3K-AKT axis, is indispensable for B cell activation (49). Importantly, FoxO1 induces autophagy in various cells and diseases including neuroblastoma (50, 51). FoxO1 knockout in osteoblasts hinders the binding of FoxO1-ATG7, leading to alleviated intracellular autophagy (52). Considering that the FoxO1 inactivation was involved via phosphorylation by the PI3K/AKT pathway, we further examined the phosphorylation of FoxO1 under PD-1 overexpression and knockdown in IBDV-infected DT-40 cells. We found that PD-1 promoted FoxO1 phosphorylation during IBDV infection. Furthermore, the phosphorylation level of FoxO1 changed upon treatment with the inhibitor LY294002 or the agonist SC79, consistent with previous studies (53). Here, we demonstrated that PD-1 promotes IBDV proliferation via the PI3K/AKT/FoxO1 pathway. Therefore, the molecular mechanism about the role of PD-1 in the PI3K/AKT/FoxO1 pathway following IBDV replication needs to be studied further.

The inhibition of autophagosome formation prevents viral DNA/RNA synthesis and subsequently affects the progress of the viral replication cycle (54). Additionally, IBDV infection enhances autophagy initiation, which is critical for promoting viral replication (21). Autophagy can serve as an antiviral mechanism and can also be exploited by viruses to evade immune surveillance. IBDV can evade immune responses by regulating the autophagy process. For instance, it can prevent the degradation of viral particles by inhibiting the fusion of autophagosomes and lysosomes. However, the specific molecular mechanisms linking the PI3K/AKT/FoxO1 pathway to IBDV-induced autophagy in B lymphocytes have not been reported. Using starvation or 3-MA treatment, we found that IBDV replication is essential for autophagy induction in DT-40 cells. Notably, PD-1 promotes IBDV replication by activating autophagy, providing replication sites for viral infection. Thus, we believe that IBDV infection activates PD-1-induced autophagy via the PI3K/AKT/FoxO1 signaling pathway to support viral infection. However, whether chicken PD-1 is a novel cellular receptor for IBDV-infected B cells still requires further research.

## 5 Conclusion

In conclusion, we found that IBDV infection upregulates PD-1 expression, which interacts with and enhances VP2 expression. Mechanistically, PD-1 induces autophagy to facilitate IBDV replication via the PI3K/AKT/FoxO1 pathway. Thus, compounds that activate or inhibit PD-1 may support or block IBDV replication. These results improve our understanding of the complex interplay between autophagy and IBDV infection as well as preliminarily identify a novel potential target for the development of universal vaccine adjuvants against IBDV.

## Data Availability

The datasets presented in this study can be found in online repositories. The names of the repository/repositories and accession number(s) can be found in the article/supplementary material.
